# The 23^rd^ AMN Congress | Integrated Pathway Management in Neurotrauma: From ER and ICU to Rehabilitation and Mental Health

**DOI:** 10.25122/jml-2026-1014

**Published:** 2026-08

**Authors:** Alexandra Gherman, Stefana-Andrada Dobran, Dafin Fior Muresanu

**Affiliations:** 1 RoNeuro Institute for Neurological Research and Diagnostic, Cluj-Napoca, Romania; 2 Department of Neuroscience, Iuliu Hatieganu University of Medicine and Pharmacy, Cluj-Napoca, Romania

## AMN: Towards an Integrated Approach to Neurotrauma Care

Over the years, the Academy for Multidisciplinary Neurotraumatology (AMN) has grown significantly as a global medical society, launching new projects and building upon its core concept of advancing neurotraumatology in research, medical care, advocacy, and education.

As the AMN expanded its scope, new developments were promoted. The **AMN INTENSIVES (Figure 1)** aims to bring greater dynamism into neurotrauma care, with successful projects such as the Hands-On Teaching Course on Acute and Chronic Stress, Sequelae and Ways of Relaxation with Focus on Autogenic Training, which opened this year’s Congress, as well as the AMN Neurotrauma Scientific Symposium “Comprehensive Approach to Traumatic Brain Injury: Emergency Care, Technological Applications, and Personalized Treatment”, hosted at University Medical Center Ho Chi Minh City (Vietnam) on June 13^th^ 2026.

**Figure 1:**
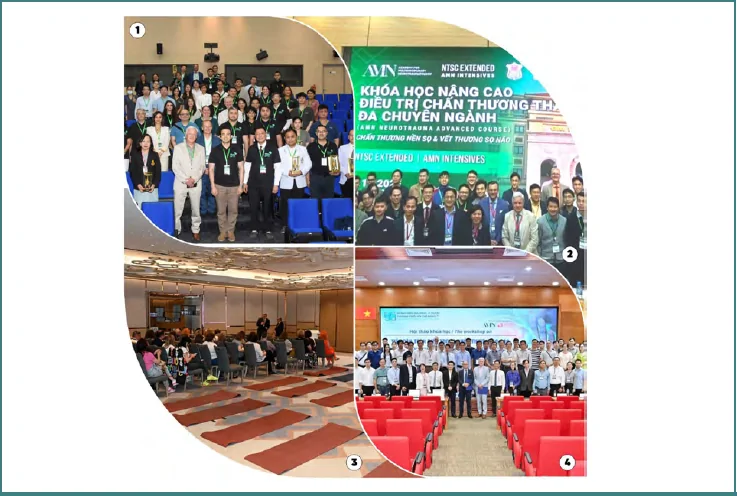
The AMN Intensives editions: (1) NTSC Extended | AMN Intensives, 1^st^ Edition - Thailand (July 3^rd^, 2025 | Bangkok), (2) AMN Neurotrauma Advanced Course – Skull Base & Penetrating Brain Trauma NTSC Extended | AMN Intensives - 2^nd^ Edition, Vietnam (November 4^th^, 2025 | Hanoi), (3) Hands-On Teaching Course on Acute and Chronic Stress, Sequelae and Ways of Relaxation with Focus on Autogenic Training - Turkey (May 28^th^, 2026 | Istanbul), and (4) AMN Intensives 4^th^ Edition - AMN Neurotrauma Scientific Symposium Comprehensive Approach in Traumatic Brain Injury: Emergency - Technological Application - Personalized Treatment - Vietnam (June 13^th^, 2026 | Ho Chi Minh City)

Continuing the journey of Neurotrauma Treatment Simulation Center (NTSC) Vienna, a core new concept, **NTSC Extended**, launched in 2025 as an instrumental item of the AMN INTENSIVES. Following two successful iterations in Bangkok (Thailand) and Hanoi (Vietnam) **(Figure 1)**, this one-day intensive format aims to train specialists in neurotrauma management, following the principle of the ‘*Patient Pathway*’ towards recovery from the early acute care through to long-term neurorehabilitation.

The AMN continues to advance and become an international hub for experts from all disciplines involved in the care of neurotrauma patients, including neurologists, neurosurgeons, intensive care specialists, psychiatrists, psychologists, rehabilitation specialists, and many more.

## The 23^rd^ AMN Congress: Integrated Pathway Management in Neurotrauma: From ER and ICU to Rehabilitation and Mental Health

On 28-30 May 2026 in Istanbul (Türkiye), a landmark event paved the way towards improving the status of neurotrauma care worldwide. The 23^rd^ Congress of the Academy for Multidisciplinary Neurotraumatology once again united multidisciplinary experts from all fields related to neurotrauma under the theme “*Integrated Pathway Management in Neurotrauma: From ER and ICU to Rehabilitation and Mental Health*” **(Annex 1)**.

Annex

Coordinated by Prof. Dr Fior-Dafin Mureșanu (Romania), AMN Secretary-General, Prof. Johannes Vester (Germany), AMN President, and Prof. Dr Volker Hömberg (Germany), Chairman of the AMN Scientific Program Committee, with the support of the local organizing committee, composed of Prof. Dr Emre Adıgüzel, Dr. Belgin Erhan, Dr. Evren Yaşar, Dr. Berke Aras, and Prof. Dr Hakan Ekmekci **(Figure 2)**, the Congress united over 40 international high-level speakers and reached a record of 1,000 online and on-site participants. With over 24 countries represented, the scientific event truly stands as an example of international collaboration and continues the journey of the AMN towards building a comprehensive and multidisciplinary approach to neurotrauma.

**Figure 2:**
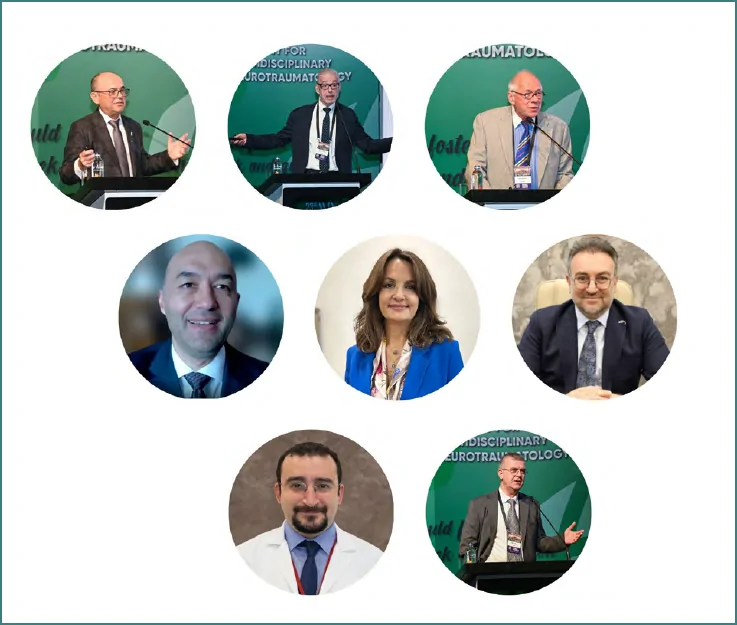
23^rd^ AMN Congress Coordinators and Local Organizing Committee (from left to right): first row - Prof. Dr Fior-Dafin Mureșanu (Romania), AMN Secretary-General, Prof. Johannes Vester (Germany), AMN President, and Prof. Dr Volker Hömberg (Germany), Chairman of the AMN Scientific Program Committee; second row - Prof. Dr Emre Adıgüzel, Dr. Belgin Erhan, Dr. Evren Yaşar; third row - Dr. Berke Aras, and Prof. Dr Hakan Ekmekci, all from Türkiye

## Hands-On Teaching Course on Acute and Chronic Stress, Sequelae and Ways of Relaxation with Focus on Autogenic Training

The first day of the AMN Congress brought forward the dynamic ‘Hands-On Teaching Course on Acute and Chronic Stress, Sequelae and Ways of Relaxation with Focus on Autogenic Training” **(Figure 3)**, coordinated by Prof. Dr Max Hilz, Adjunct Professor of Neurology at Icahn School of Medicine at Mount Sinai, New York, NY, USA. Held on May 28^th^, the course welcomed almost 40 participants from multiple specialities, to learn to improve stress resilience and stress management.

**Figure 3:**
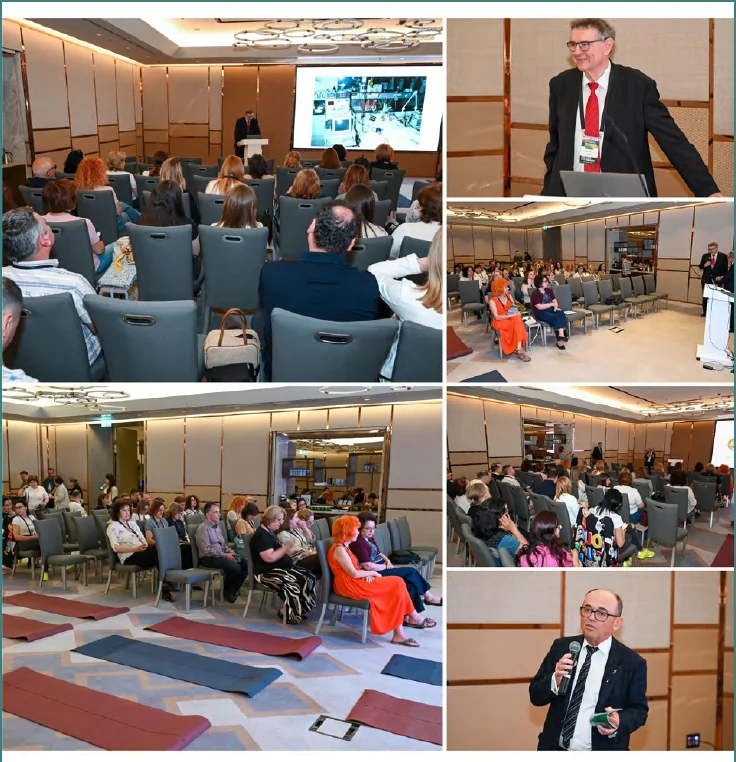
The Hands-On Teaching Course on Acute and Chronic Stress, Sequelae and Ways of Relaxation with Focus on Autogenic Training, coordinated by Prof. Dr Max Hilz (top right). Prof. Dr Max Hilz was introduced to the course participants by Prof. Dr Fior-Dafin Mureșanu (bottom right), AMN Secretary-General.

Structured in three parts, the course began with an introduction to the physiology of acute and chronic stress, with particular emphasis on the role of the autonomic nervous system in regulating organ function, especially the cardiovascular system.

Exploring the broad impact of stress on health, the course addressed a variety of complications ranging from syncope, cardiac arrhythmias, and Takotsubo syndrome, to gastrointestinal dysfunction, sexual dysfunction, and anxiety, with particular attention given to psychological changes associated with stress in individuals with chronic diseases and patients with a history of traumatic brain injury. It then explored various methods for alleviating stress, and introduced the participants to the various components of Autogenic Training and its beneficial effects for both physical and psychological consequences of anticipatory stress before engaging in practical training sessions designed to experience the technique firsthand.

The *Hands-on Teaching Course* familiarized participants with the steps of Autogenic Training and encouraged them to regularly practice and actively engage with it for reducing the negative impact of stress and alleviating physical and psychological complaints.

## The 23^rd^ AMN Congress: The Patient’s Journey Towards Recovery

With a dynamic scientific program **(Figure 4)**, the Congress sessions followed the patient pathway from acute care towards neurorehabilitation by tackling the following main themes:

- Modern Neuroscience Approaches for Neurotrauma: From Barrier Protection to Novel Therapeutics;
- Acute Management/Critical Care: Overlooked Acute Complications in TBI: Early Management as a Determinant of Long-Term Outcome;
- Diffuse Axonal Injury as a Translational Model for Cerebroprotection in Neurotrauma;
- Surgical Brain Injury (SBI): The Unavoidable Cost of Life-Saving Neurointerventions;
- The Invisible Aftermath of Neurotrauma: Neuropsychiatric Sequelae and Their Impact on Recovery.

**Figure 4:**
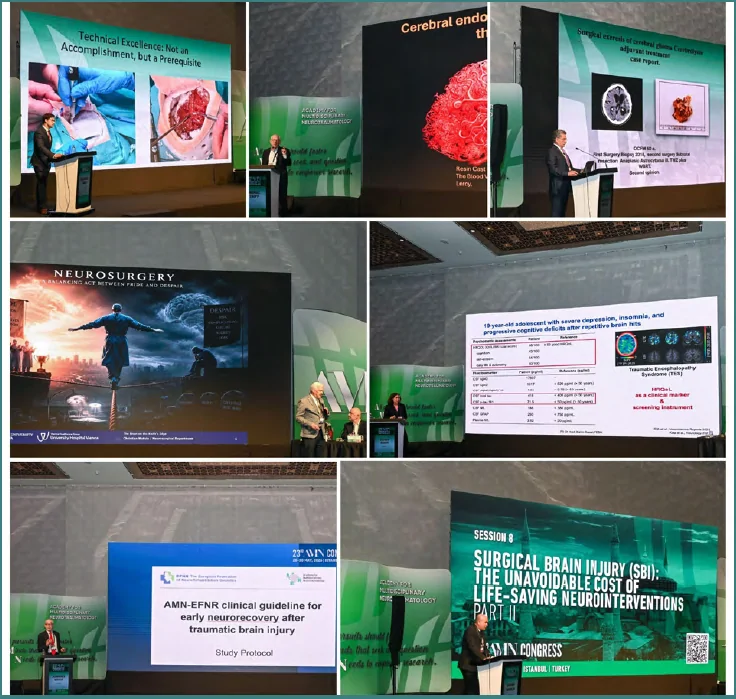
Presentations from the 23^rd^ AMN Congress

Bringing together leading experts in neurotraumatology, the opening Presidential Session explored current challenges and future directions in neurotrauma management and neurorehabilitation. The role of the AMN in advancing education, research, and integrated care was highlighted during a Special Keynote Lecture, while following presentations explored emerging perspectives in post-TBI rehabilitation and post-traumatic Parkinsonism. The session introduced another central pillar of AMN activity currently under development: the PRESENT Registry – a comprehensive multidisciplinary TBI directory that aims to improve healthcare delivery and offer a greater understanding of traumatic brain injury.

As part of the interactive format, two debates brought into the spotlight contrasting discussions on the necessity of robust basic care compared to advanced technological perspectives in “Debate 1: *High-Tech Neurotrauma Care vs. Robust Pathway Basics: What Truly Improves Long-Term Outcomes in LMICs?*” as well as on the importance of early rehabilitation during “Debate 2: *Is Early Rehabilitation a Luxury or a Necessity in Neurotrauma Pathways?*” **(Figure 5)**. Using an interactive response system, participants were encouraged to take short quizzes during each presentation, engaging in lively talks on the arguments presented.

**Figure 5:**
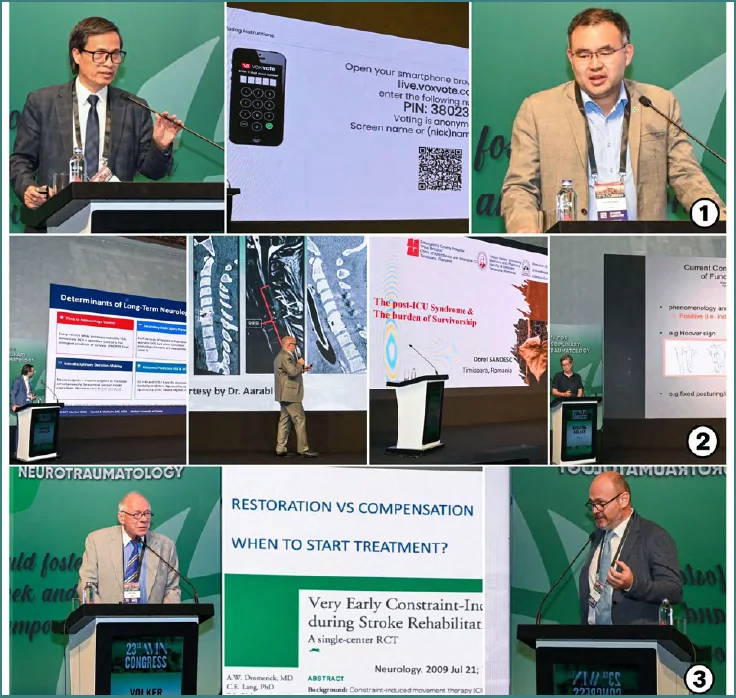
(1) Debate 1: High-Tech Neurotrauma Care vs. Robust Pathway Basics: What Truly Improves Long-Term Outcomes in LMICs?, (2) Interactive Panel Discussion – Polytrauma and Neurotrauma: Sequencing Decisions That Shape Long-Term Outcomes, (3) Debate 2: Is Early Rehabilitation a Luxury or a Necessity in Neurotrauma Pathways?

**Figure 6:**
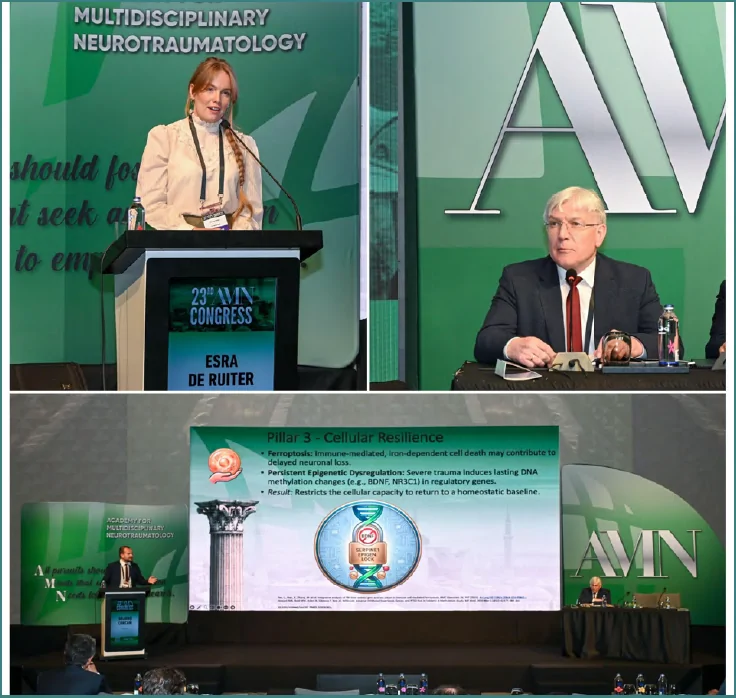
Young Neurotrauma Network Session, with Ms. Esra de Ruiter, Prof. Dr Mihail Gavriliuc, and Mr. Dragos Corcan

In the spirit of multidisciplinary approaches, an interactive Panel Discussion **(Figure 5)** addressed polytrauma and neurotrauma, and the decisions that shape long-term outcomes, uniting perspectives of specialists in acute care, neurosurgery, and rehabilitation.

The final session was dedicated to promoting ongoing research projects in traumatic brain injury, from clinical guidelines to molecular profiling, highlighting current advances and emerging directions in the field.

Continuing the tradition from the past AMN Congress, several speakers were awarded as Highlight of the Congress **(Annex 1)**, reflecting the impact of their presentations *on the scientific program of the congress and overall development of the scientific event*.

A special address during the opening session was dedicated to the memory of Prof. Dr Nilda Turgut. Prof. Turgut, Head of the Department of Neurology at Tekirdağ Namık Kemal University (Türkiye), was a valued colleague, close friend, and longstanding collaborator of the Academy for Multidisciplinary Neurotraumatology, whose passing in 2024 was a great loss to the neurotrauma community. In recognition of her exceptional contributions and enduring commitment to the Academy, AMN posthumously awarded her Honorary Membership **(Annex 1)**.

## Young Neurotrauma Network

A newly introduced concept, the **AMN Neurotrauma Network**, aims to develop a community for experts, young specialists, neurotrauma patients, and caretakers with an integrated and effective approach to traumatic brain injury (TBI) care. It addresses three main goals (1) creating a special hub for patients recovering from TBI, (2) supporting young specialists from all domains within neurotraumatology to share their knowledge and experience and receive support from senior members, and (3) encouraging professional development and patient engagement through mentorship and guidance from experienced AMN members.

As a first step in initializing this new pursuit, the special session of the Congress “**Young Neurotrauma Network**” united three different perspectives from young professionals, experienced educators building up new generations, and patients with neurotrauma on the road to recovery.

As community and collaboration stand at the core of the AMN, we wish to see this pursuit expand and create a hub for integrated exchange and support within the AMN Neurotrauma Network in a shared environment of mentorship, collaboration, and patient-centered care.

## New Educational Perspectives

The ***AMN Educational Insights*** and ***History of Neurotrauma* (Figure 7)** are the two new projects launched during this years' congress intended to reach out to and attract the wider audience — medical specialists, students, patients, care-givers — present in the online environment.

**AMN Educational Insights** is a series of short videos showcasing several aspects of neurotraumatology, from introductory concepts to consequences of TBI, risk groups, diagnosis and imaging, and neurorehabilitation. The format will allow for accessible, focused learning that can enable members to engage with topics in a concise and practical manner.

On a similar note, **AMN History of Neurotrauma** follows the trail of already published material presenting, in a story-like approach, the advancements in neurotrauma care, from the oldest prehistoric times up to modern day. This limited series will immerse watchers in the fascinating progress, showcasing neurotraumatology as one of the oldest and most interesting branches of medicine.

Both series launched on the AMN Social Media channels and will serve as an educational tool for all members, from patients to specialists.

Similar to previous years, a series of **video interviews (Figure 7)** with distinguished faculty members will highlight their perspectives on the role of the congress, advances in their respective fields, and highlight their views on future collaboration with the AMN. The interviews will also be available on the AMN Social Media channels.

**Figure 7:**
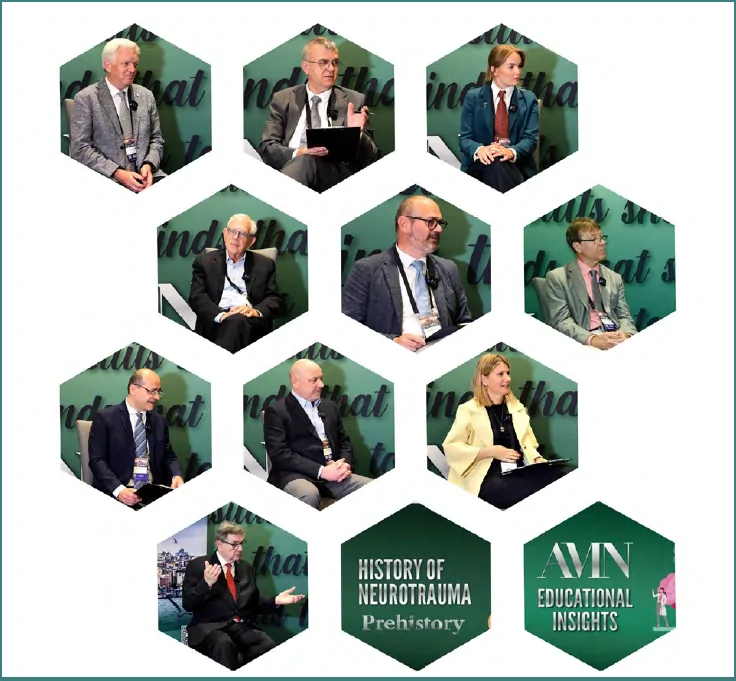
Photos from the interviews with some of the speakers from the 23^rd^ AMN Congress and History of Neurotrauma & AMN Educational Insights logos

**Figure 8:**
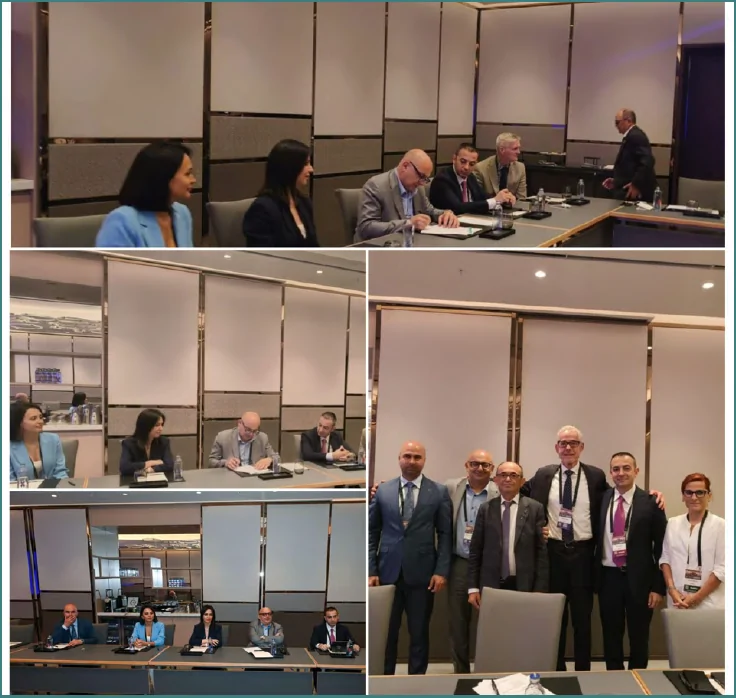
Official Ceremony for Signing the Official Acknowledgement of the Azerbaijan Focus Group and the Memorandum of Understanding with Azerbaijan Neurosurgical Association (ANEA)

## AMN Focus Groups: Advancing Collaboration in Neurotraumatology

A newly launched concept of the AMN, the AMN Focus Groups (FGs) represented a central element of this year’s Congress, with not only a dedicated special session that brought experts from Romania, Egypt, and Thailand, but also with launching the first AMN Focus Group - Focus Group Azerbaijan (FG Az).

The Focus Groups aim to bring together experts at the country level and enhance the entire dynamic of AMN, incorporating national and international partnerships, cooperation, or collaborations between AMN and scientific bodies or companies related to the field of neurotraumatology.

Coordinated by Prof. Dr Rovshan Khalilzada, from the Azerbaijan Medical University (Baku), and uniting seven members with different specialties, namely Dr. Mubariz Rahimov, Dr. Konul Ahmadova, Dr. Parvin Akbarov, Dr. Leyla Merjan, Dr. Azer Zeynalov, and Dr. Sait Merjan, FG Az aims to establish a National Epidemiological Database and a National Trauma Registry, identify disease burden and treatment gaps, develop national recommendations, improve multidisciplinary trauma care, participate in the formation of health policy and the allocation of resources, and of scientific research and international cooperation.

We wish the best of luck to our colleagues in this endeavor and look forward to the formation of more focus groups in different countries. We are proud to see this initiative grow and expand.

Moreover, building upon the relationship with Azerbaijan, the AMN signed a Memorandum of Understanding with the Azerbaijan Neurosurgical Association (ANEA) to strengthen collaboration between the two institutions, promote the AMN and ANEA as leading societies for the management of traumatic brain injuries globally, as well as improve education and advocacy globally.

## Conclusions

The 23^rd^ AMN Congress continued the journey of the Academy in reaching its goals for a more integrated, multidisciplinary approach to neurotrauma. Standing the test of time, the AMN Congresses evolve every year and, on each edition, bring forward new concepts and academic instruments adherent to the pillars of the AMN as well as enhance and encourage the access of specialists, patients and care-givers to its global multidisciplinary network.

We await you next year, at the 24^th^ AMN Congress in Tashkent, Uzbekistan, on 17-20 June **(Figure 9)**, to build upon the success of all previous editions and support us in advancing the multidisciplinary neurotrauma care and foster the Academy for Multidisciplinary as a benchmark in the scientific environment.

**Figure 9:**
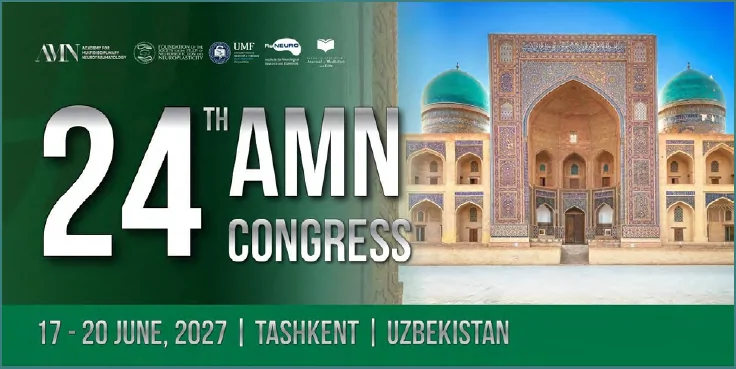
The announcement of the 24^th^ AMN Congress in Tashkent, Uzbekistan

