# Supplementary material for: The 23rd AMN Congress | Integrated Pathway Management in Neurotrauma: From ER and ICU to Rehabilitation and Mental Health
Source: J Med Life. 2026 Aug;19(8):604–12. doi: 10.25122/jml-2026-1014 (PMC13674052; doi:10.25122/jml-2026-1014)
Supplement: Supplementary file 1 [file JMedLife-19-604-s001.pdf]

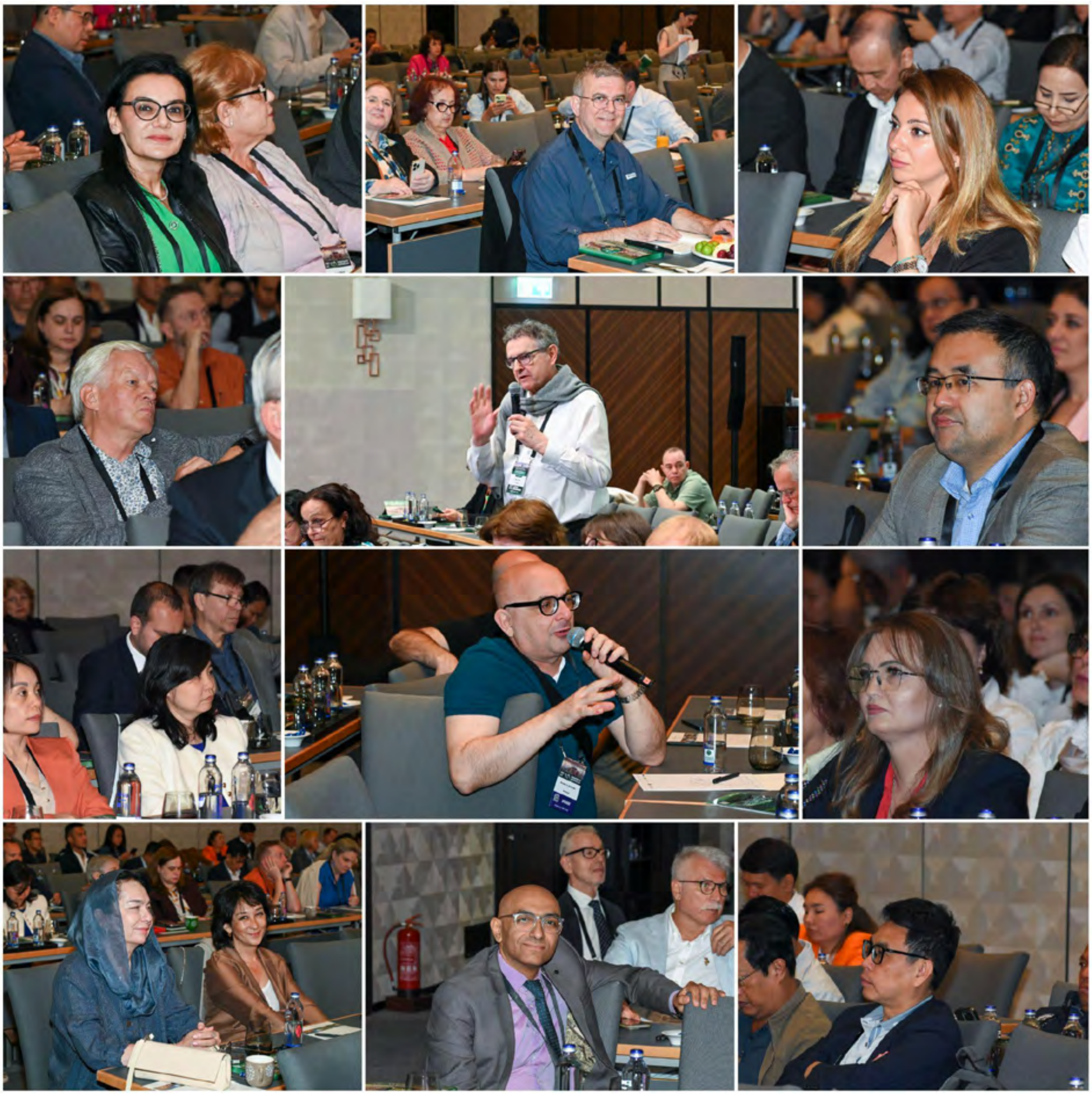

A. Photos of speakers and participants at the 23rd AMN Congress

© 2026 by the authors. This article is an open access article distributed under the terms and conditions of the Creative Commons Attribution (CC BY 4.0) license.

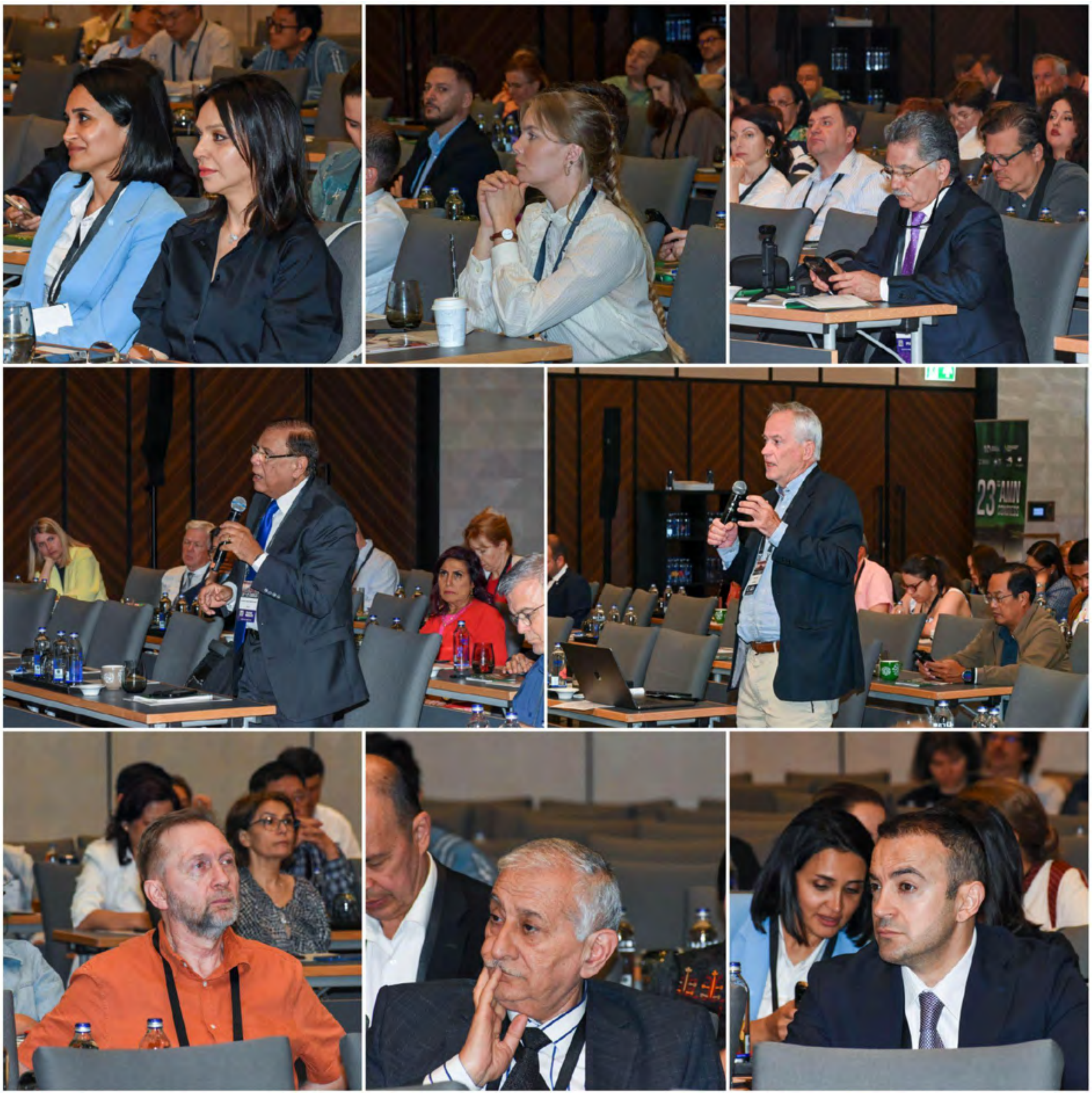

B. Photos of the speakers and participants from the 23rd AMN Congress

© 2026 by the authors. This article is an open access article distributed under the terms and conditions of the Creative Commons Attribution (CC BY 4.0) license.

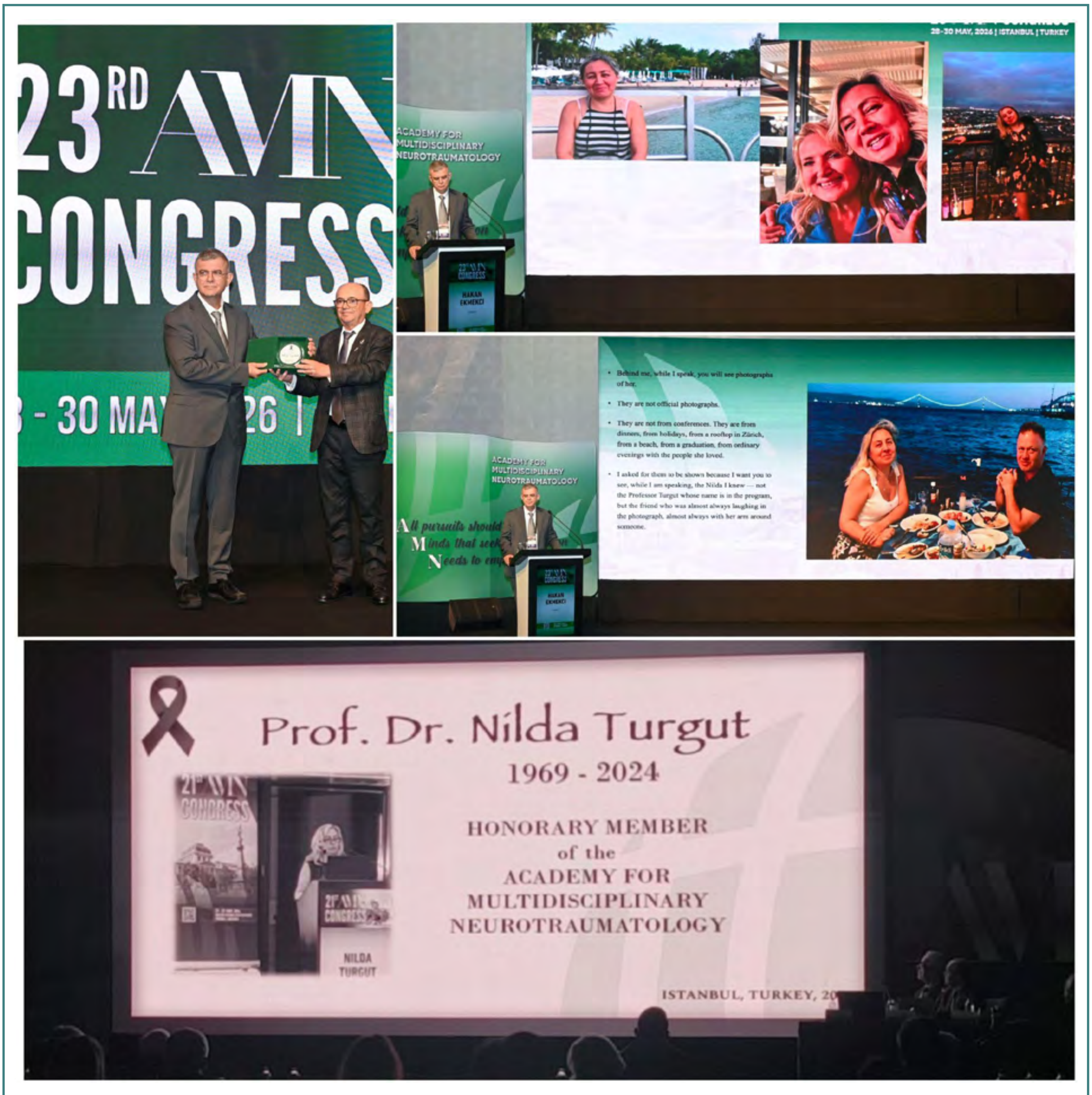

C. Special Address of Prof. Dr Hakan Ekmekci during the Opening Session of the Congress: In Memoriam Prof. Dr Nilda Turgut

© 2026 by the authors. This article is an open access article distributed under the terms and conditions of the Creative Commons Attribution (CC BY 4.0) license.

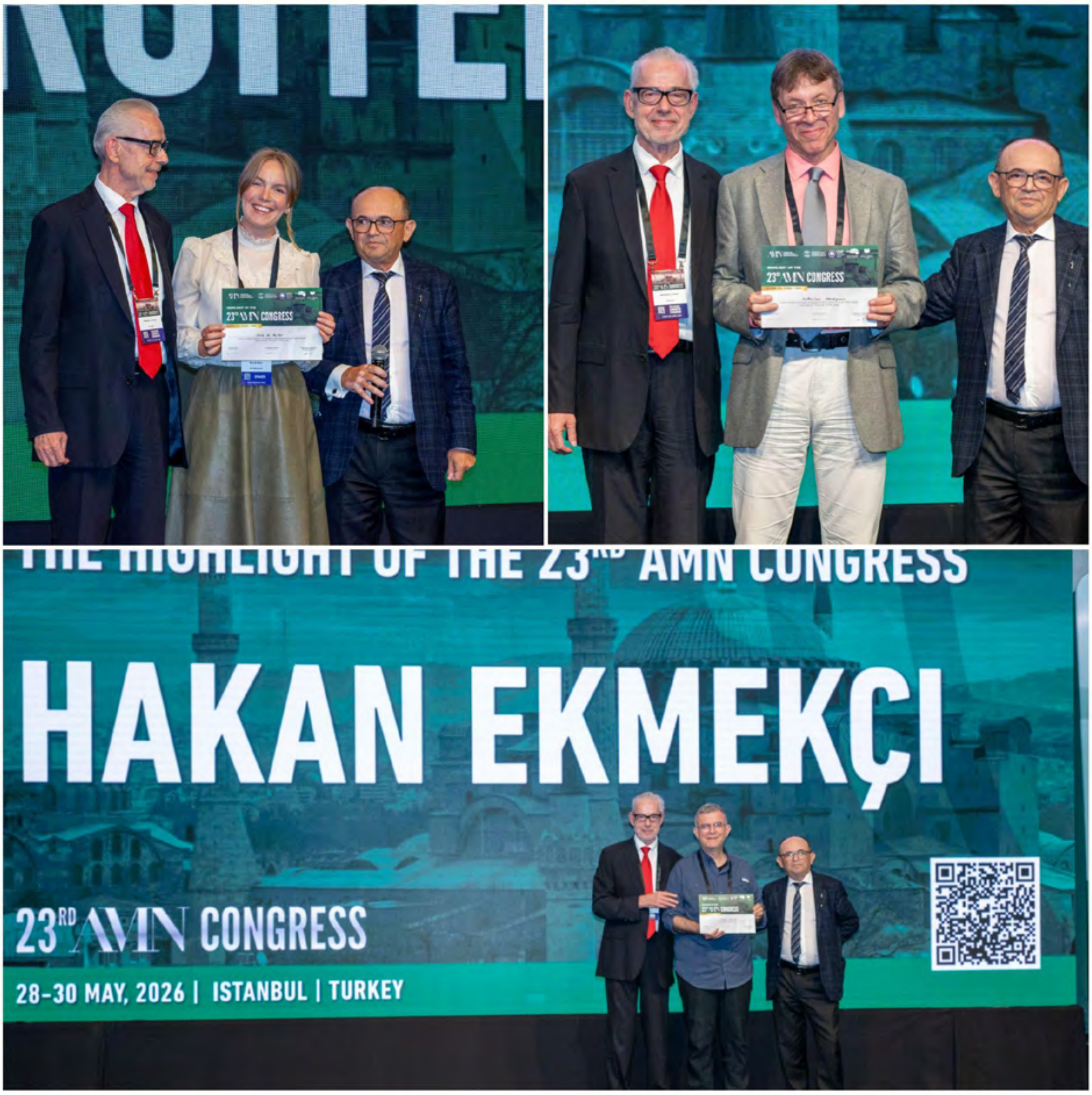

D. The highlights of the 23rd AMN Congress: Ms. Esra de Ruiter (the Netherlands), Dr. Octavian Henegariu (Romania), and Prof. Dr Hakan Ekmekci (Türkiye)

© 2026 by the authors. This article is an open access article distributed under the terms and conditions of the Creative Commons Attribution (CC BY 4.0) license.

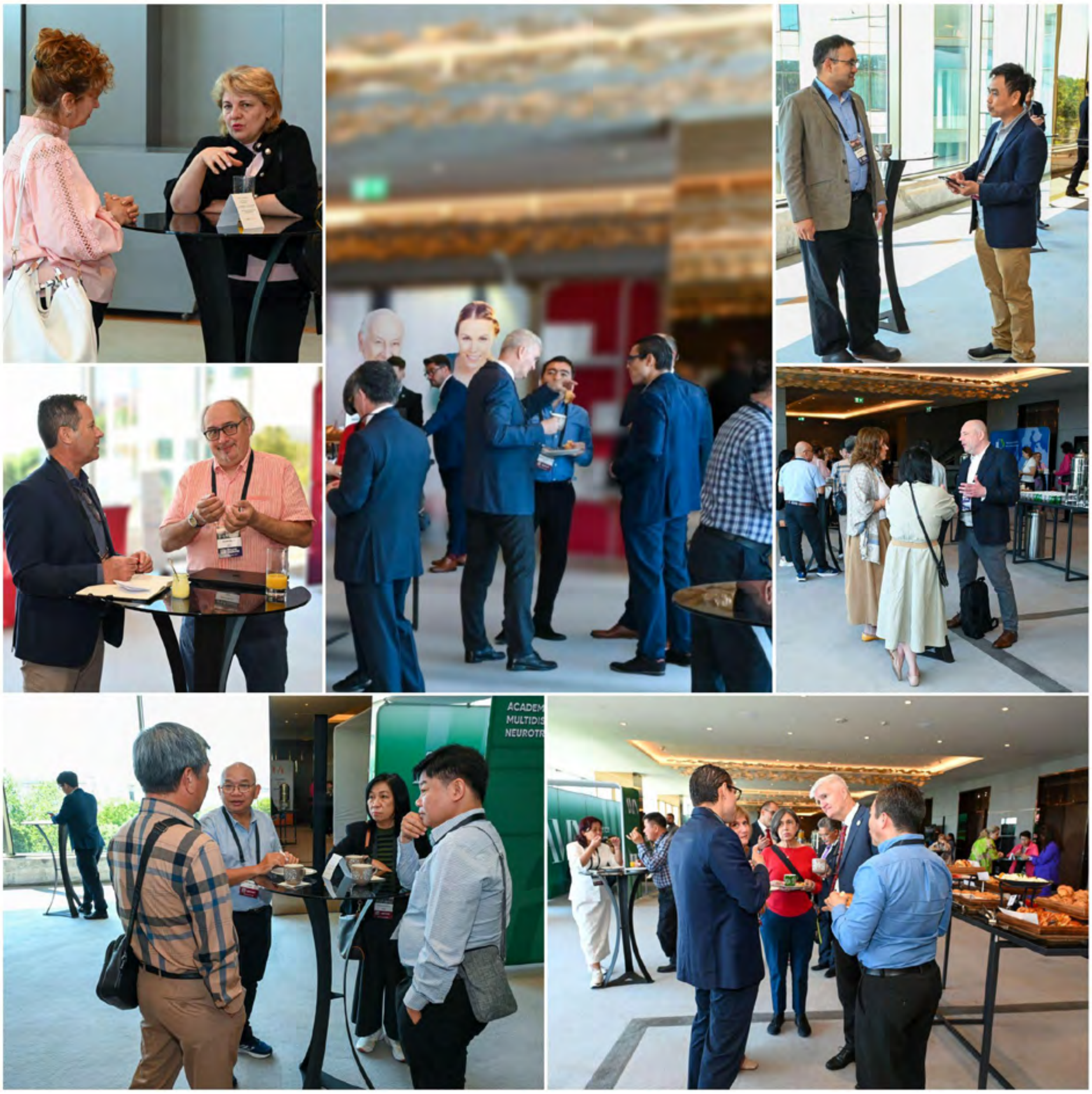

E. Photos during networking and breaks at the 23rd AMN Congress

© 2026 by the authors. This article is an open access article distributed under the terms and conditions of the Creative Commons Attribution (CC BY 4.0) license.

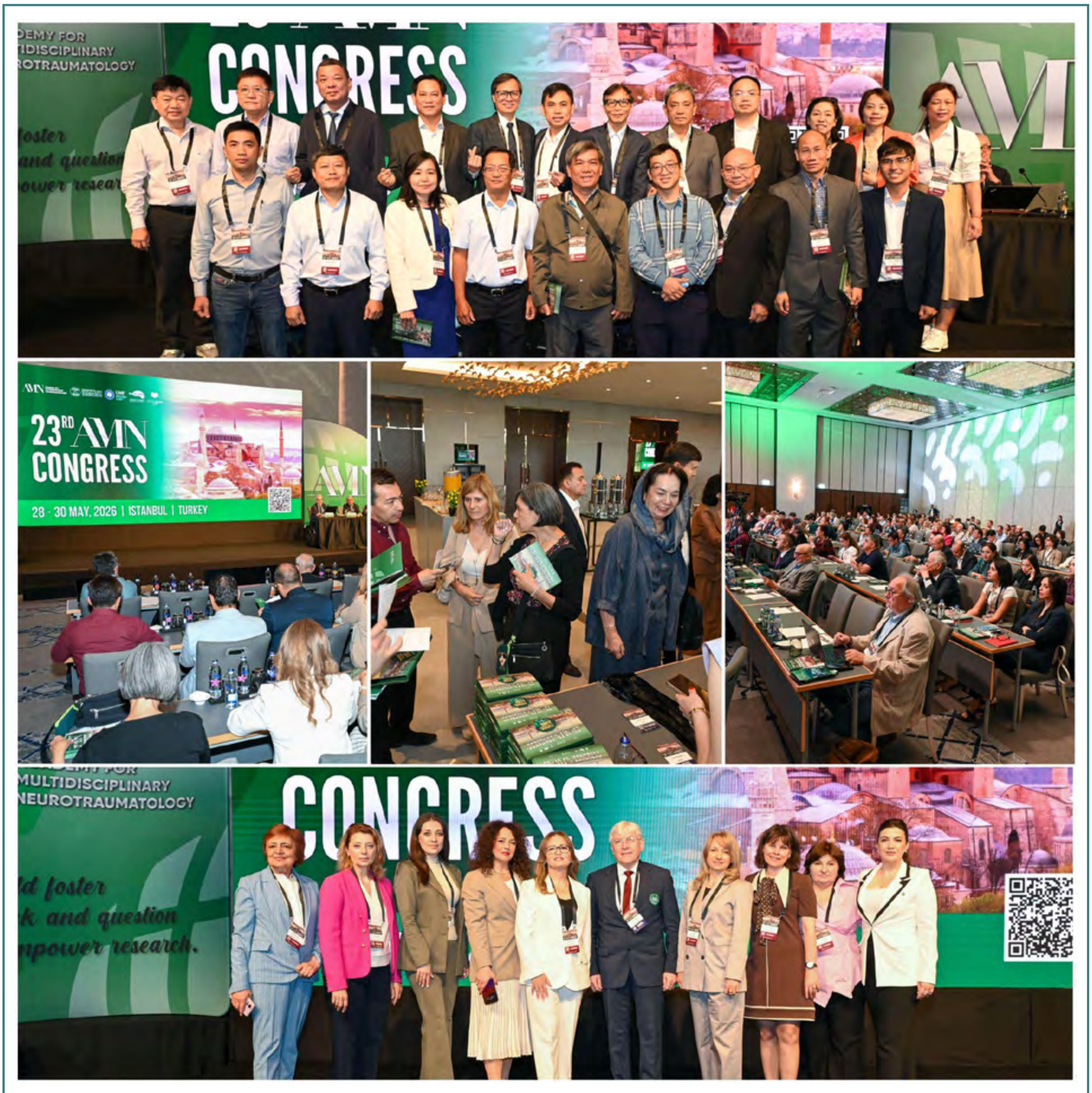

F. Group photos and photos of the participants and speakers from the AMN Congress

© 2026 by the authors. This article is an open access article distributed under the terms and conditions of the Creative Commons Attribution (CC BY 4.0) license.

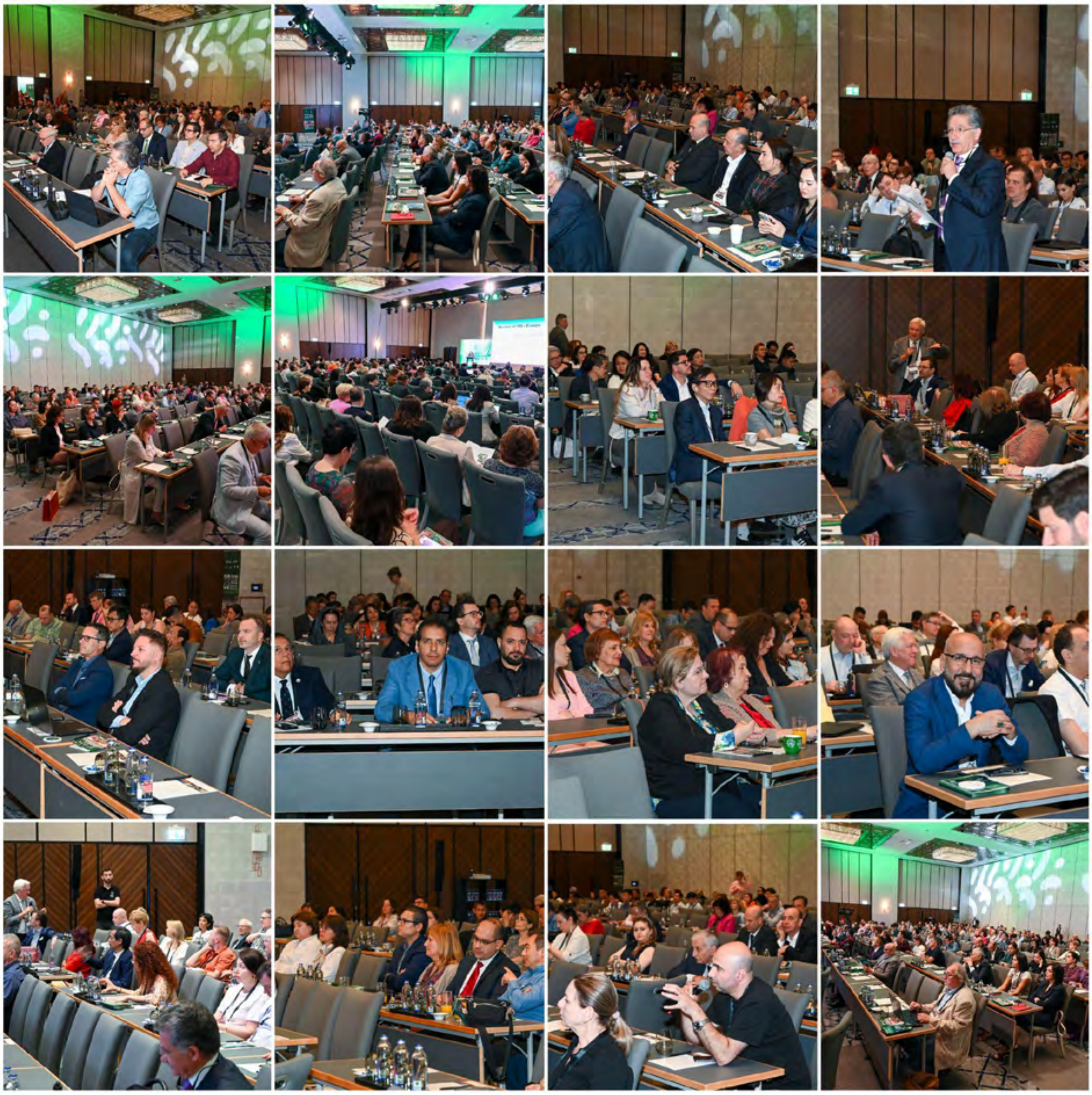

G. Photos from the 23rd AMN Congress

© 2026 by the authors. This article is an open access article distributed under the terms and conditions of the Creative Commons Attribution (CC BY 4.0) license.

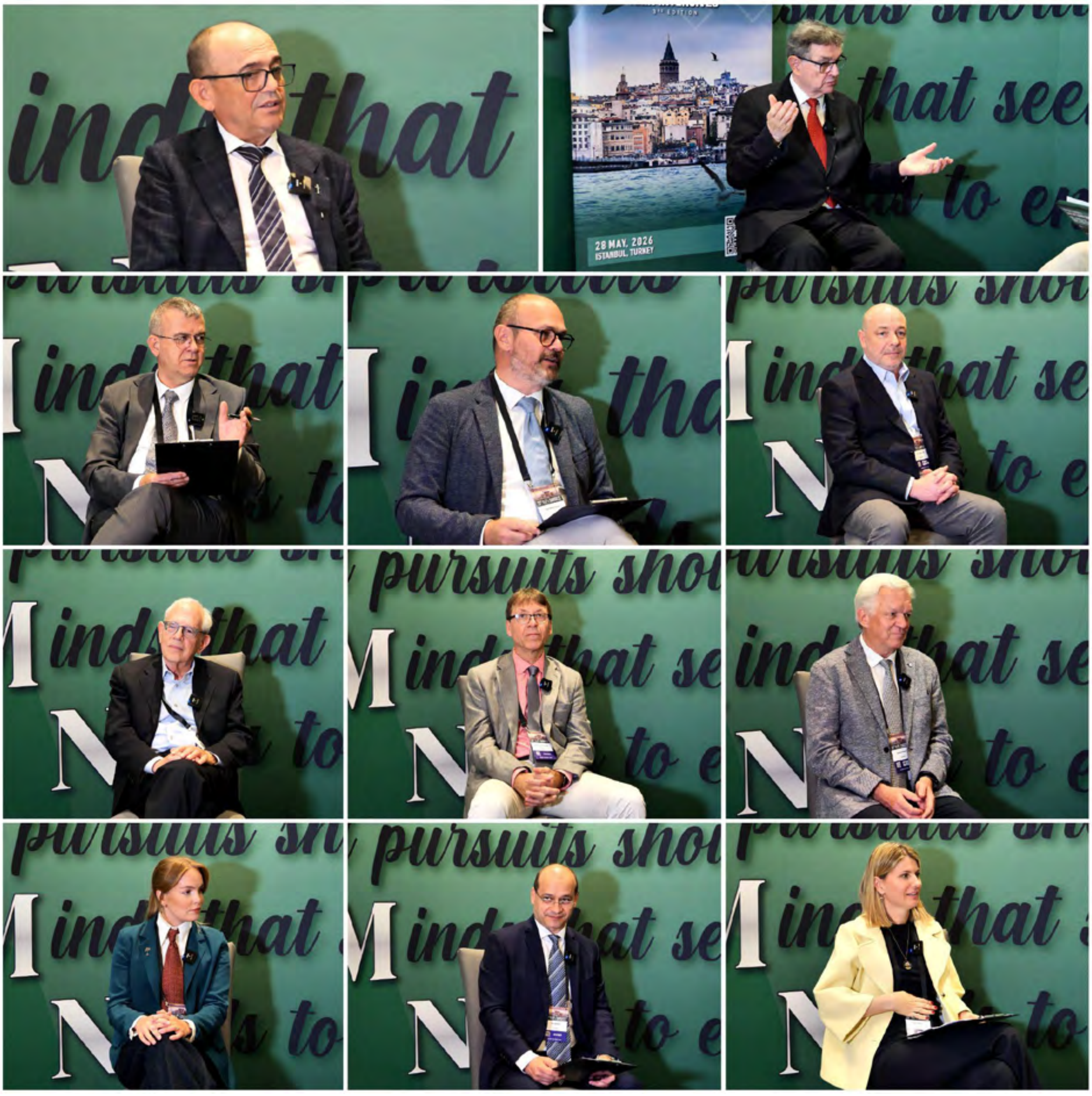

H. Photos from the interviews conducted during the 23rd AMN Congress

© 2026 by the authors. This article is an open access article distributed under the terms and conditions of the Creative Commons Attribution (CC BY 4.0) license.
